# The influence of ergonomic factors on the work conditions of paramedics during the rescue of trauma patients in an ambulance

**DOI:** 10.1016/j.heliyon.2025.e42370

**Published:** 2025-01-29

**Authors:** Daniel Celiński, Sylwia Bęczkowska, Iwona Grabarek, Katarzyna Grzybowska, Zuzanna Zysk, Tadeusz Miłowski, Krzysztof Marek Mitura, Sławomir Dariusz Szajda

**Affiliations:** a Department of Emergency Medical Service, Medical University of Warsaw, Poland; b Division of Transport, Warsaw University of Technology, Poland; c Department of Emergency Medical Service, Collegium Medicum, University of Warmia and Mazury in Olsztyn, Poland; d Independent Public Health Care Center RM-MEDITRANS Emergency Station and Sanitary Transport in Siedlce, Poland

**Keywords:** Paramedic, Ambulance, Occupational risk factors, Body movement, EMG, Injury

## Abstract

**Introduction:**

The risk factors encountered in the work of paramedics include ergonomic, physical, psychological, and biochemical factors. This study identifies discomforts experienced by paramedics during medical procedures in ambulances, emphasizing their negative impact on musculoskeletal load and forming the basis for ergonomic and organizational recommendations.

**Methods:**

The study employed non-invasive MyoMotion and EMG methods to measure musculoskeletal strain during typical stationary and transit medical procedures.

**Results:**

Preliminary results showed musculoskeletal overloads resulting from paramedics assuming forced positions during medical procedures on trauma patients. Some of these positions, especially while the ambulance was in motion, were characterized by a high risk of injury.

**Conclusions:**

An ergonomic ambulance interior can help reduce the risk of injuries during medical procedures.

## 1 Introduction

The profession of a paramedic primarily involves providing professional assistance in cases of sudden threats to health or life. In their daily work, paramedics must make quick and accurate decisions, requiring skills and knowledge across various fields of medicine. A paramedic's duties include assessing the patient's condition and taking appropriate medical actions to maintain their life and health [1]. Paramedics work in diverse environmental conditions and locations, so the hazards associated with their work vary in nature and influence their occupational risk to different extents. The risk factors encountered in the work of paramedics fall into four main categories: physical, chemical, biological, and psychosocial, with some researchers adding a fifth category of ergonomic factors. The primary adverse effect of ergonomic factors targets the musculoskeletal system, particularly the spine. Medical procedures performed on patients often require lifting and maintaining forced, unnatural positions [[2], [3], [4]].

Emergency medical services (EMS) are considered a type of work where employees are regularly exposed to physical exertion and danger [[3], [4], [5], [6]]. Therefore, ensuring ergonomic working conditions for paramedics should be a priority as they affect the level of work comfort and minimize the likelihood of injuries. Statistics indicate an increasing shortage of paramedic staff worldwide. In the United States and Germany, the annual turnover of paramedics is around 10 % [[7], [8], [9]]. Overall, 54 % of paramedics consider leaving EMS teams within the next year, with 46 % dissatisfied with their working conditions [5].

Due to the nature of work in an ambulance, it is challenging to precisely define the paramedic's workplace. There are virtually no restrictions on where paramedics provide assistance, especially when aiding trauma patients. Time spent in the ambulance cabin performing medical procedures constitutes about 44 % of the active work dedicated to patient care [10]. According to the definition, an ambulance is a means of transport dispatched to the scene of a sudden illness or accident, intended for providing assistance and transporting the sick or injured from the incident site to the hospital. It is often used for medical and inter-hospital transports. Ambulances are operated by specially trained rescue teams and are part of the emergency response system [1,3,11,12].

Since most paramedic activities are performed in the ambulance, our study focused on the paramedic's workstation inside the ambulance [11,13,14]. The development of musculoskeletal disorders among paramedics is attributed to muscle fatigue associated with prolonged execution of various medical procedures, including those requiring relatively low muscle activation. Paramedic fatigue is most often caused by the overload of the musculoskeletal system. Therefore, reducing load and fatigue in working conditions can be a critical element in eliminating or mitigating existing musculoskeletal disorders [15,16]. Thus, the use of appropriate methods for assessing load and muscle fatigue is essential [17].

In our study, we proposed the following hypothesis: identifying the discomforts experienced by paramedics while working in the ambulance is fundamental to developing recommendations regarding the spatial structure of the ambulance and organizational guidelines. The authors’ previous experiences [18] have enabled the development of a methodology for obtaining data on paramedic work in the ambulance regarding musculoskeletal fatigue and the load associated with forced positions during medical procedures on trauma patients.

## 2 Material and methods

### 2.1 Data acquisition methodology

The main challenge in obtaining data that reflect the load and fatigue of the musculoskeletal system, as well as posture during medical activities for the ergonomic evaluation of paramedics, is the necessity to limit the methods to non-invasive ones that do not interfere with the subject's body. Additionally, these methods should collect data in real-time without restricting the paramedic's ability to perform their work. In this case, two methods that meet the above criteria were found useful. The first is the surface electromyography (sEMG) method, which enables the recording of the electrical activity of muscles using surface electrodes and thus provides information about muscle fatigue. The second allows the registration of the kinematics of individual body segments during medical activities, offering the possibility to assess the degree of strain in the paramedic's body position during typical procedures. The kinematic motion studies were conducted while stationary, whereas EMG measurements were taken during ambulance movement. The authors limited this article to presenting selected results recorded during medical procedures performed on a trauma patient. The number of participants in a study is crucial for the generalizability of the results. This study was of a pilot nature and aimed to identify key ergonomic issues related to the work of paramedics. The results obtained from a single participant allowed for the development of a methodology that will be applied to a larger number of participants in future studies. One of the main challenges in studies of this type is the availability of ambulances and paramedics. In Poland, as in many other countries, ambulances are in constant use, and their availability for research purposes is limited. Each study required renting an ambulance for a specific period, which involved additional costs and logistical arrangements, as well as affecting the availability of paramedics, who are typically engaged in life-saving work in the field. As a result, conducting studies with a larger number of participants under real-world conditions is a challenging logistical endeavor and requires extensive preparation, which is currently underway. The goal of the presented study, as mentioned multiple times, was different, and that is why we decided to focus on a single, qualified paramedic.

**Fig. 1:**
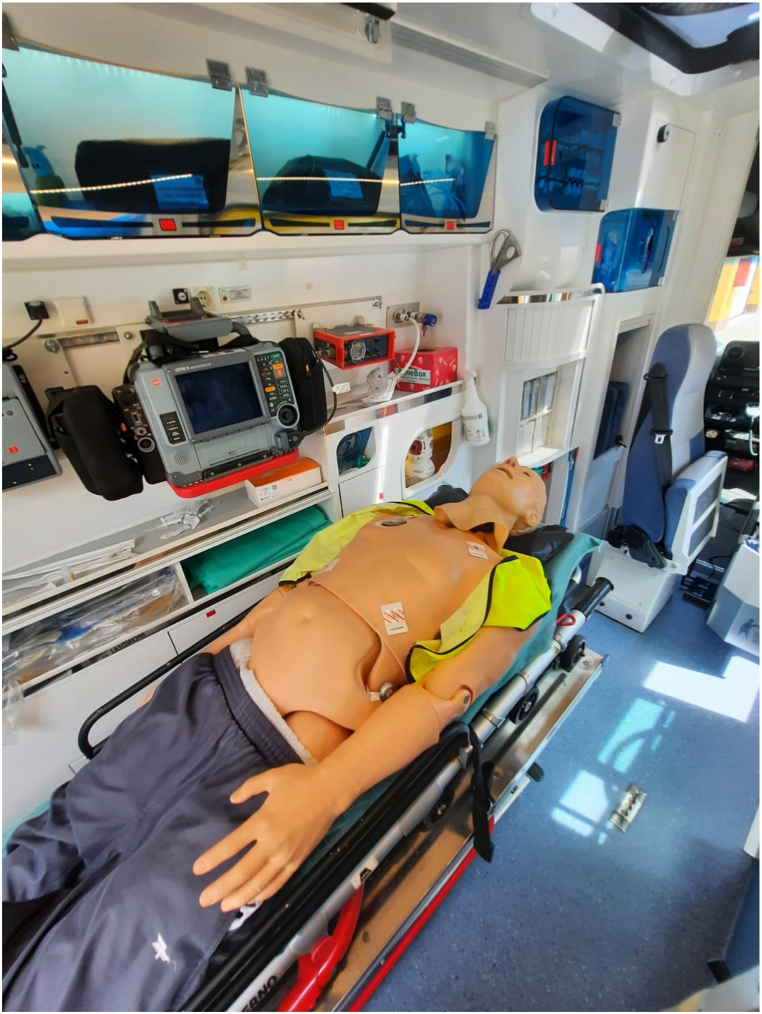
Test bed - the interior of an ambulance with full medical equipment and a medical simulator on a stretcher.

The study was conducted in an ambulance of the Independent Public Healthcare Institution RM-MEDITRANS Emergency Medical Services and Sanitary Transport Station in Siedlce (Siedlce Ambulance Service). The participant was a male paramedic, aged 39 years, with a height of 178 cm and weight of 80 kg. He had no history of musculoskeletal disorders, and his last occupational health examination, conducted on June 29, 2023, did not reveal any medical contraindications to his work. The paramedic performed selected medical activities both stationary and in motion. Seventeen procedures most commonly performed in ambulances were examined, based on data collected from experienced paramedics. This paper presents results for only five procedures that are most frequently performed when assisting trauma patients. The test station was located in a Mercedes ambulance standardized according to the standards in force at the Siedlce Ambulance Service. Medical procedures were performed by an experienced paramedic on a medical simulator in the form of high-fidelity simulation (Fig. 1). All procedures were conducted in a moving ambulance under real-world conditions. The pilot nature of the study, involving a paramedic, allowed for the identification of discomforts experienced during the work of EMS team members.

The study (9/2022) was conducted with the approval of the Research Ethics Committee of the Warsaw University of Technology on November 23, 2022.

### 2.2 Medical procedures

#### 2.2.1 Analyzed medical procedures

The study presents results for the following procedures: immobilization of the lower limb (P1), immobilization of the upper limb (P2), thermos-insulation (P3), stopping of lower limb hemorrhage (P4), and trauma examination (P5). The paramedic performed the procedures in both standing and seated positions, often adopting a bent posture. Special wire splints, known as Kramer splints, were used for immobilizing the limbs. Immobilization was carried out according to Pott's principle and included at least two adjacent joints, below and above the fracture site, without attempting to realign the fracture. This prevents the bone fragments from shifting relative to each other and reduces pain at the fracture site [19].

Ensuring thermal comfort involves thermal insulation using a thermal blanket, commonly known as an “emergency blanket.” This procedure entails tightly wrapping the entire patient in the blanket. This is used to prevent hypothermia or overheating of the patient. The thermal blanket is designed to prevent heat loss and protect against excessive overheating [19].

The procedure for “stopping of lower limb hemorrhage”, was performed by the paramedic in a standing position. During this procedure, the paramedic compressed the patient's femoral artery with their right leg while simultaneously applying a dressing to the bleeding site. To achieve this, the paramedic had to adopt a forced body position, lifting their right leg and leaning towards the wound [19].

The trauma examination was carried out by the paramedic in a standing position according to current guidelines. This examination involves assessing the patient's condition by evaluating vital signs and searching for any injuries that pose a life-threatening condition. During this procedure, mainly the senses of sight and touch are used to detect any abnormalities. After assessing the vital signs, the paramedic sequentially examines the following body parts: the head, neck, chest, abdomen, pelvis, lower and upper limbs, back, and buttocks [19].

**Fig. 2:**
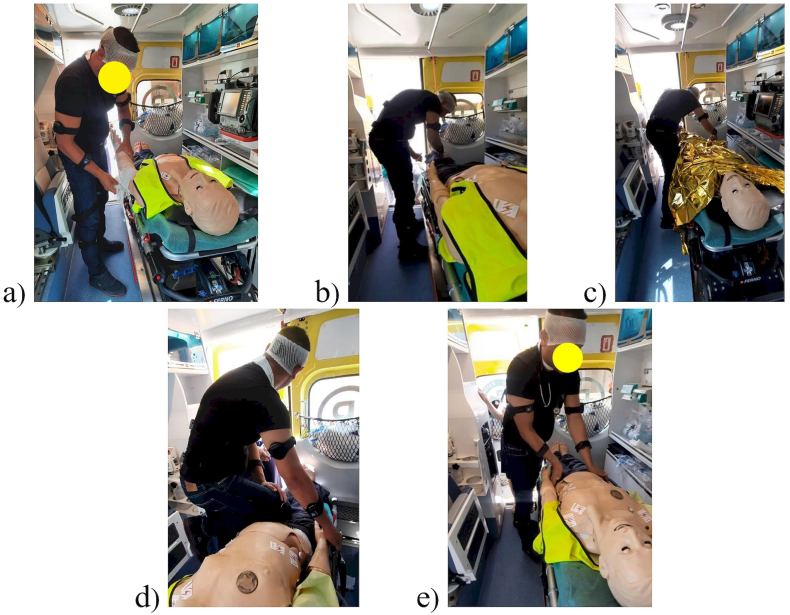
The paramedic during: a) immobilisation of the upper limb, b) immobilisation of the lower limb, c) thermo-insulation, d) stopping of lower limb hemorrhage, e) trauma examination.

Fig. 2 shows examples of positions adopted by the paramedic during the medical procedures.

#### 2.2.2 The most common medical procedures used in trauma, using the example of the eastern Mazovia emergency medical service

**Fig. 3:**
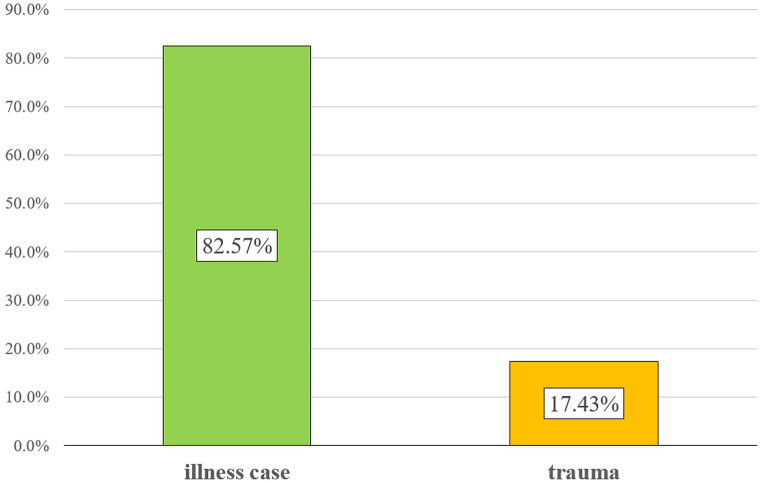
List of interventions made by Emergency medical team in the Eastern Mazovia area in 2022.

The Independent Public Healthcare Institution RM-MEDITRANS Emergency Medical Services and Sanitary Transport Station in Siedlce provides healthcare services in the field of medical rescue and health promotion in the eastern Mazovia region (Poland). The EMS region, under the jurisdiction of the Siedlce Emergency Service, covers the areas of the following counties: Siedlce, Sokołów, Łosice, Mińsk, Garwolin, and Węgrów. In this region, there are 24 emergency medical teams, including 5 specialized and 19 basic teams [20]. Based on statistical data from the Command Support System of the National Medical Rescue Service, which is a nationwide register of EMS interventions, it was determined that the number of interventions in the area under the jurisdiction of the Siedlce Emergency Service in 2022 was 39,705, averaging 1654 trips per EMS team annually, which is about 5 interventions per day. In 17.43 % (n = 6920) of these cases, the incidents were caused by injuries. Fig. 3 shows the percentage distribution of interventions in 2022, categorized by type of incident.

The Independent Public Healthcare Institution RM-MEDITRANS Emergency Medical and Sanitary Transport Station in Siedlce codify the medical services they perform based on the International Classification of Medical Procedures (ICD-9) to classify and organize the surgical, diagnostic, therapeutic, and even billing procedures they perform. This allows for the assignment of appropriate codes to procedures related to the use of health services.

Table 1 presents selected medical procedures that the EMS teams from eastern Mazovia performed during trauma-related interventions. The compilation covers the period from January 1 to December 31, 2022.

**Table 1:** Medical procedures performed on-site in 2022 in the eastern Mazovia region for patients with trauma.

Procedure	Trauma	
	n	%
89.79 Physical examination – other	6295	90.97 %
89.71 Patient assessment to determine the course of action and decision to initiate or withhold emergency medical procedures	5452	78.79 %
89.07 Subjective examination (history taking)	3713	53.66 %
89.540 Monitoring of basic vital functions	2136	30.87 %
93.5000 Treatment of head injuries	906	13.09 %
93.5004 Treatment of upper limb injuries	361	5.22 %
93.5022 Immobilization of the lower limb	360	5.20 %
93.5020 Immobilization of the upper limb	323	4.67 %
93.521 Application of cervical collar	271	3.92 %
93.5017 Immobilization using a spine board	254	3.67 %
93.5006 Treatment of lower limb injuries	240	3.47 %
93.57 Application of wound dressing – other	227	3.28 %
93.5001 Treatment of facial injuries	143	2.07 %
93.542 Application of Kramer splint	63	0.91 %
93.5002 Treatment of nasal injuries	56	0.81 %
93.5005 Treatment of upper limbs injuries	46	0.66 %
93.5007 Treatment of lower limbs injuries	29	0.42 %
93.549 Application of other immobilizing splint	28	0.40 %
93.573 Application of hydrogel dressing	25	0.36 %
93.5021 Immobilization of the upper limbs	22	0.32 %
93.5008 Treatment of chest injuries	21	0.30 %
93.5011 Treatment of abdominal injuries	18	0.26 %
93.5023 Immobilization of the lower limbs	13	0.19 %
93.5009 Treatment of anterior chest injuries	13	0.19 %
93.5010 Treatment of posterior chest injuries	11	0.16 %
93.5015 Treatment of ear injuries (external, middle, and/or inner ear)	4	0.06 %
93.5012 Treatment of pelvic injuries	4	0.06 %
93.564 Tactical tourniquet	4	0.06 %
93.5018 Immobilization using a vacuum mattress	3	0.04 %
93.5019 Immobilization using Kendrick Extrication Device (KED)	3	0.04 %
93.572 Hemostatic dressing – tactical gauze for hemorrhage control with reduced activation time	3	0.04 %
93.5016 Treatment of ear injuries (external, middle, and/or inner ear)	2	0.03 %
93.5025 Pelvic stabilization belt	2	0.03 %

Based on the data, we see that the most common procedures performed on trauma patients included trauma examinations (anamnesis and physical examination). Following these were procedures related to trauma treatment and immobilization of the limbs. The described procedures do not include thermal comfort measures because this procedure is not listed in the ICD-9 procedure classification. Nonetheless, it is performed according to guidelines in situations where there is a risk of hypothermia or hyperthermia in the patient, and in patients with extensive trauma, it is recommended to use it to combat traumatic hypothermia.

### 2.3 Registration of motion kinematics during medical activities

#### 2.3.1 Studied ranges of motion of anatomical joint angles

The experimental study involved the measurement of 31 anatomical angles of body elements (joints) on both sides of the body. The angles studied are shown in Table 2.

**Table 2:** Summary of anatomical angles of body segments studied.

L.p.	List of body parts
1.	Cervical Flexion
2.	Cervical Lateral – RT
3.	Cervical Axial – RT
4.	Lumbar Flexion
5.	Lumbar Lateral – RT
6.	Lumbar Axial – RT
7.	Thoracic Flexion
8.	Thoracic Lateral – RT
9.	Thoracic Axial – RT
10.	Elbow Flexion LT
11.	Elbow Flexion RT
12.	Shoulder Total Flexion LT
13.	Shoulder Total Flexion RT
14.	Shoulder Flexion LT
15.	Shoulder Flexion RT
16.	Shoulder Abduction LT
17.	Shoulder Abduction RT
18.	Shoulder Rotation – out LT
19.	Shoulder Rotation – out RT
20.	Hip Flexion LT
21.	Hip Flexion RT
22.	Hip Abduction LT
23.	Hip Abduction RT
24.	Hip Rotation – out LT
25.	Hip Rotation – out RT
26.	Knee Flexion LT
27.	Knee Flexion RT
28.	Knee rotation – out LT
29.	Knee rotation – out RT
30.	Knee abduction LT
31.	Knee abduction RT

#### 2.3.2 Measuring equipment

The study used the MyoMotion system by Noraxon. This system combines wireless data transmission technology and inertial sensors to assess any 3D movement, with software that enables both data recording and comprehensive analysis.

#### 2.3.3 Research procedure

The determination of anatomical angles was carried out according to the principles of the neutral/zero medical method. It is assumed that in a standing person, all joints are in the zero position, even if there is a deviation from the geometric zero angle. For example, the geometric angle at the ankle joint is 90°, while the anatomical angle is zero degrees.

**Fig. 4:**
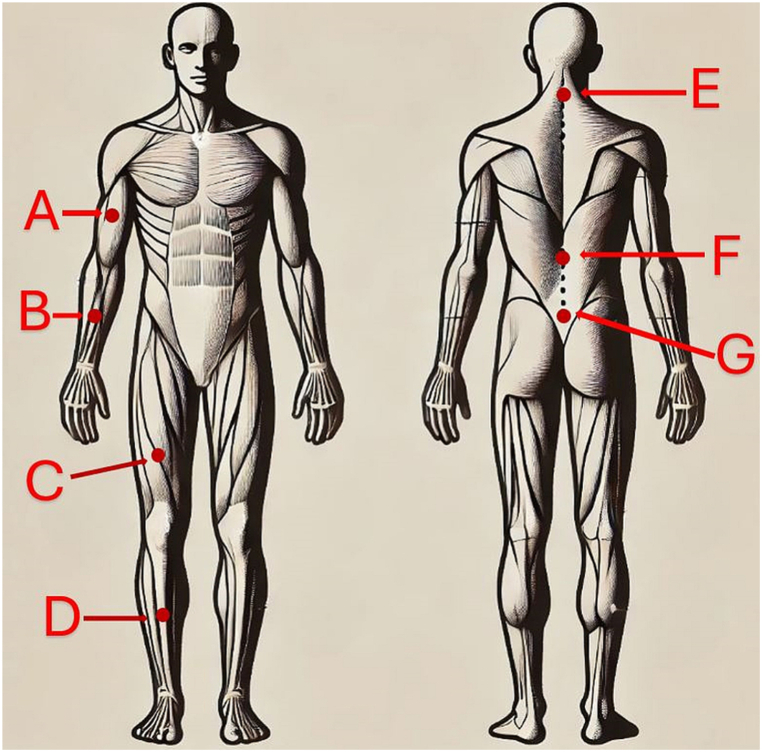
Positioning of sensors on the paramedic: front and rear view.

Each sensor has labels for the X, Y, and Z axes. All sensors were placed on body segments in such a way that, in the standing position, the X-axis vector on the sensor's label pointed vertically along the axis of the gravitational force. Inertial sensors were placed on the body of the subject paramedic to record accelerations in accordance with the MyoMotion protocol. The placement of the sensors is shown in Fig. 4 and detailed in Table 3.

**Table 3:** Summary of sensor locations.

Symbol	Body model sensor location
A	Upper Arm (RT & LT)
B	Forearm (RT & LT)
C	Thigh (RT & LT)
D	Shank (RT & LT)
E	Pelvis
F	Lower Spine
G	Upper Spine

After placing the sensors in the correct positions, they were calibrated. During calibration, the subject had to stand still in a specified position (upright position, feet hip-width apart, arms along the torso at shoulder width). It is assumed that the angle for the calibration position is zero, while negative values indicate a direction to the left and lateral flexion, whereas positive values always indicate a direction to the right. After calibration, the paramedic could proceed with the individual medical procedures (P1, P2, P3, P4, P5). There are negative and positive values for measuring angle changes, with the calibration position set to zero.

#### 2.3.4 Evaluation criteria

The selected evaluation criteria for individual joints were adopted according to values for the normal range of motion and are presented in Table 4. Range of Motion (ROM) refers to the joint's ability to go through the full spectrum of movements. The range of motion of a joint can be passive or active. Each joint has a normal range of ROM values, although each person has a different ability to achieve it. It is important to note that not every individual necessarily has the same ranges. Older individuals typically have a reduced range of motion compared to younger individuals, and those with chronic injuries may also have diminished ranges of motion. Range of Motion is the overall motion used in a movement and can be specified by linear or angular motion of the body segments. The values were taken for the age range corresponding to labor force participation of 18–65 years. Two ranges are given in the table, as these ranges vary slightly in the various sources available. These are averaged values for both populations, although in reality the range of motion in some joints in women is greater than in men.

**Table 4:** Normal ranges of motion.

Joint/segment	Plane of motion	Type of motion			Degrees [standard ISOM]
		(−)		(+)	
Cervical Spine	Sagittal	Extension	0	Flexion	40^0^–0 – 40^0^
	Frontal	Left lateral bending	0	Right lateral bending	45^0^–0 – 45^0^
	Rotation	left axial rotation	0	Right axial rotation	50^0^–0 – 50^0^
Thoracic Spine∗	Sagittal	Extension	0	Flexion	25^0^–0 – 35^0^
	Frontal	Left lateral bending	0	Right lateral bending	25^0^–0 – 25^0^
	Rotation	left axial rotation	0	Right axial rotation	30^0^–0 – 30^0^
Lumbar Spine∗	Sagittal	Extension	0	Flexion	15^0^–0 – 50^0^
	Frontal	Left lateral bending	0	Right lateral bending	20^0^–0 – 20^0^
	Rotation	left axial rotation	0	Right axial rotation	5^0^–0 – 5^0^
Lumbar-thoracic Spine	Sagittal	Extension	0	Flexion	30^0^ – 0- 85^0^ (35^0^–0 – 85^0^)^a^
	Frontal	Left lateral bending	0	Right lateral bending	30^0^–0 -30^0^ (45^0^–0 – 45^0^)^a^
	Rotation	Left axial rotation	0	Right axial rotation	45^0^–0 – 45^0^ (35^0^–0 – 35^0^)^a^
Hip	Sagittal	Extension	0	Flexion	15^0^–0 – 125^0^
	Frontal	Abduction	0	Adduction	45^0^–0 – 25^0^
	Rotation	External rotation	0	Internal rotation	45^0^–0 – 40^0^

Adopted from: ISOM (International Standard for Orthopedic Measurement).

a Range of Motion [21], Learn muscles [22].

### 2.4 Recording of muscle tone during medical activities

#### 2.4.1 Muscles tested

**Fig. 5:**
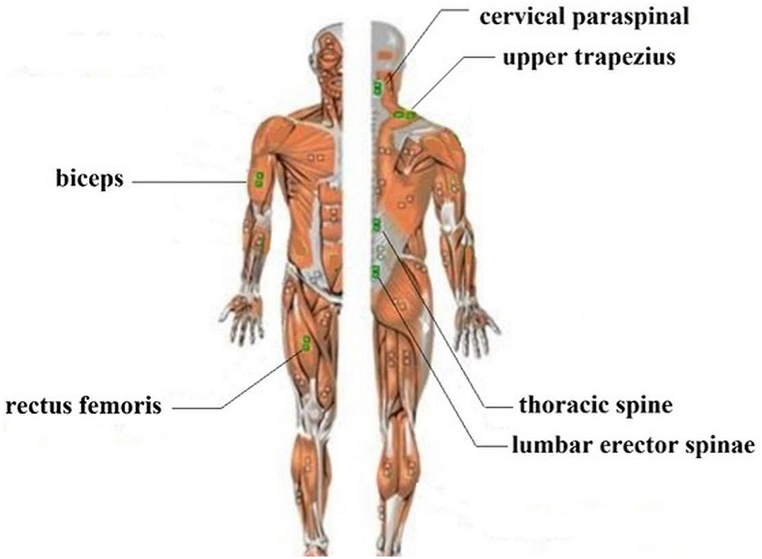
Muscles tested.

The experimental study involved the measurement of 11 muscles. The muscles tested are shown in Table 5 and Fig. 5.

**Table 5:** List of muscles tested.

No.	Latin name of muscle
1	CERVICAL PS RT
2	CERVICAL PS LT
3	UPPER TRAP. RT
4	UPPER TRAP. LT
5	BICEPS BR. RT
6	BICEPS BR. LT,
7	THORACIC ES RT,
8	THORACIC ES LT
9	LUMBAR ES RT,
10	LUMBAR ES LT,
11	RECTUS FEM. LT

#### 2.4.2 Measuring equipment

EMG signal was recorded using a device from Noraxon. Measurement control was managed via computer. The EMG signal was recorded with the Noraxon MR software version 3.10.64. The EMG sampling frequency was 1500 Hz. The bandwidth of the Noraxon device ranges from 10 to 500 Hz.

#### 2.4.3 Research procedure

**Fig. 6:**
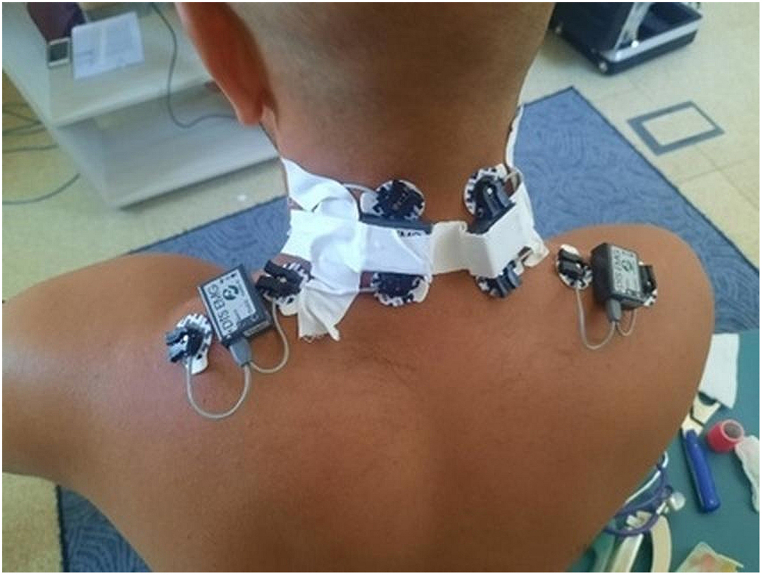
Placement of measurement sensors.

The placement of electrodes and measurement sensors on the action indicator was in accordance with the parameter guidelines in the SENIAM program (Surface ElectroMyoGraphy for the Non-Invasive Assessment of Muscles). After attaching the sensors for each muscle, the EMG signal was recorded at rest (relax: rel) and during muscle activity under conditions similar to the reference signal (ref). The ratio of the reference signal to the relax signal allowed for the determination of the quality of the EMG signal, while the reference signal served as a benchmark for the signal recorded during the medical procedure (so-called EMG signal normalization). Fig. 6 shows the placement of sensors measuring signals from the trapezius and paraspinal muscles of the cervical region. The duration of the measurement corresponded to the duration of the procedure, and measurements were taken during the movement of the ambulance.

#### 2.4.4 Parameters analyzed

The analysis of the experimental research results was performed based on the parameters of the EMG signal. These parameters are determined in the time domain (RMS amplitude) and in the frequency domain (average MPF frequency, determined based on the Fourier transform).

An important indicator of muscle activity is the amplitude of the EMG signal. The amplitude of the EMG signal can be analyzed based on the root mean square (RMS) amplitude. The method for determining the RMS parameter is described by formula (1). The RMS parameter is determined from segments of the signal (so-called windows) of a specified length.

(1) $$RMS=\sqrt{\frac{\sum_{i=1}^{n}X_{i}^{2}}{n}}$$

Where: n – the number of conducting control (window length); Xi – the value of the i-th cause [23].

#### 2.4.5 Evaluation criteria

The criteria applied for EMG evaluation stem from the physiological processes occurring in muscles under static load. During static muscle work, the inhibition of blood flow increases with the amount of force exerted. The requirements for the duration of muscle contraction depending on the level of muscle contraction force relative to its maximum capability are presented in Table 6. It is assumed that muscle tension above 20 % of the maximum force for a given muscle will indicate abnormal static muscle tension resulting from a forced or uncomfortable body position during the performance of the examined procedures. The 20 % threshold for abnormal static muscle tension is based on existing evidence (ISO/TC 159/SC 3 WG 3 N 15), which indicates that muscle tension exceeding this level correlates with increased fatigue and risk of injury during prolonged static exertion.

**Table 6:** Maximum duration of muscle contraction^a^.

Muscle contraction strength level in relation to strength (MVC) (%)	Maximum duration of muscle contraction (s)
<5	60
5–10	30
10–20	15
>20	5

a ISO/TC 159/SC 3 WG 3 N 15 Anthropometry and biomechanics.

## 3 Results

### 3.1 Range of motion of individual spinal and hip segments during medical emergency procedures

The study presents the results for 15 ranges of motion related to the cervical, thoracic, lumbar, and hip regions of the spine. The measured values for 5 medical procedures performed in an ambulance on a patient after an injury, as well as the exceedances of normal ranges of motion for each performed medical procedure, are presented in Table 7.

**Table 7:**
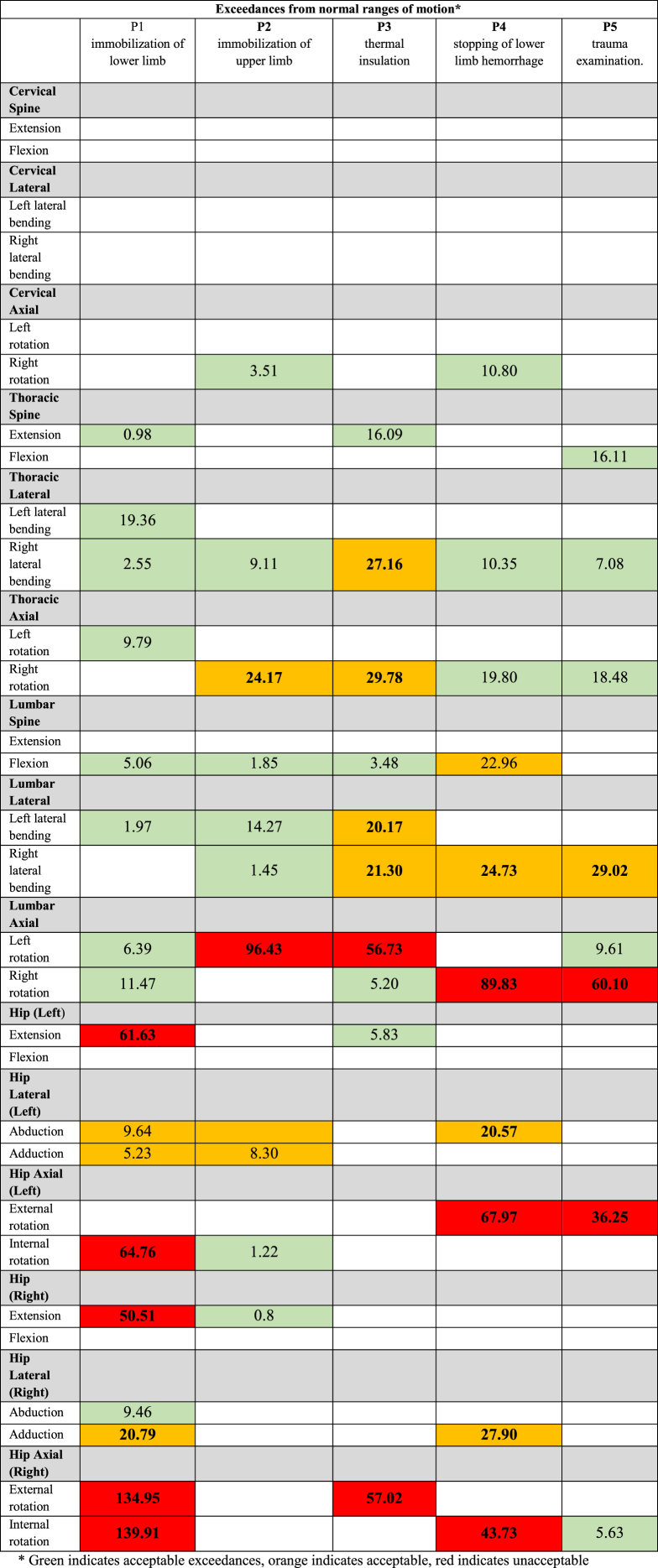
Exceeds normal ranges of movement for each medical procedure.

Assuming forced positions by the paramedic during medical procedures is a result of the movement of individual sections of the spine and hips. It was assumed that particular attention should be paid to exceedances greater than 20°. Smaller values may be insignificant and perceived as less forced, considering the fact that accepted normal values do not always reflect individual human capabilities. All these procedures are performed in forced body positions. Special attention should be paid to the range of rotational movements performed, where significant exceedances (from about 56 % to 96 %) occur in the lumbar spine, except for procedure P1. Exceedances in the range of hip joint movements were found during activities in both procedures P1 and P4.

Procedure P1, the immobilization of the lower limb, is characterized by a high load primarily on the hips, both in terms of over-extension and rotation, with the left hip being overstretched in terms of external rotation and the right hip in terms of internal rotation. A positive result is the occurrence of small range of motion exceedances in the cervical spine, which applies to all procedures. During the analysis of the signals, measurement errors were eliminated, which could have been caused, for example, by the negative impact of medical equipment operating in the ambulance cabin or sensor discharge due to prolonged measurements.

### 3.2 Muscle tension in the performance of medical emergency activities

Based on the conducted studies, an increase in tension in individual muscles was observed. Table 8 presents the resting, maximum, and average RMS values in the examined muscles, as well as the percentage value of muscle tension during the performance of medical activities on an injured patient, relative to its maximum tension.

**Table 8:**
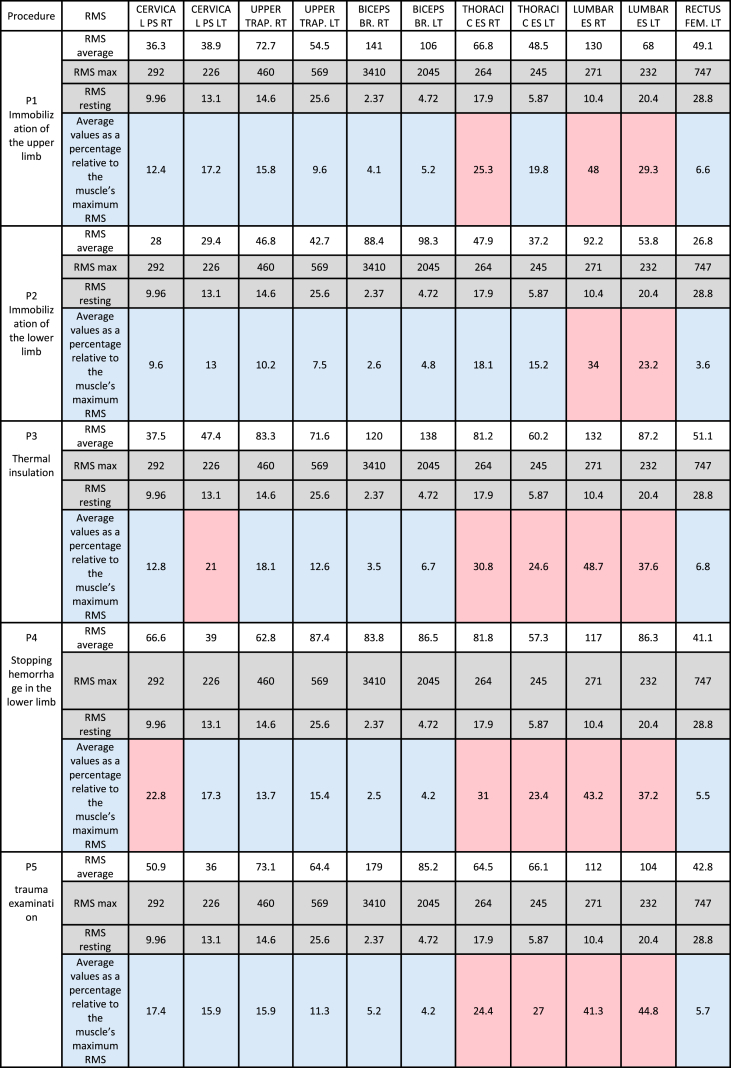
Resting RMS, average, maximum, and average values as a percentage relative to the muscle's maximum RMS during the studied procedures.

The results showed excessive muscle tension in the thoracolumbar spine. with the lowest tension during upper limb immobilization. The highest tension was recorded in the multifidus muscle, both right and left (lumbar region of the spine), and it occurred during each of the procedures. The degree of fatigue resulting from excessive muscle tensions is influenced by the duration of maintaining these positions as well as the repetitiveness of the procedures during a shift. This tension may also result from the need to stabilize the body, although the rectus femoris muscle did not show excessive tensions, it undoubtedly also influences maintaining the body in the required position. The analyzed procedures were performed by the paramedic in a standing position.

## 4 Discussion

Ensuring work safety for all employees, regardless of their occupation, is regulated by national and international laws. In recent years, the European Agency for Safety and Health at Work (EU-OSHA), the International Labour Organization, and the World Health Organization (WHO) have called for reducing health issues and emerging psychosocial risks in the workplace. Moreover, research results conducted and reported by EU-OSHA [24] confirm that physical, organizational, and psychosocial factors are the cause of many work-related health problems among employees. Currently, the growing issue of work-related musculoskeletal disorders (WRMSD) affects employees in all sectors and occupations, and its impact negatively affects health, leading to high costs for businesses and society. According to EU data, 60 % of employees with work-related health problems identify WRMSD as the most serious issue. The most frequently reported types of WRMSD include back pain, shoulder, neck, and upper limb muscle pain. Actions aimed at improving working conditions and minimizing or eliminating WRMSD-related hazards require a thorough analysis of the actual situation, i.e., identifying burdens that may contribute to musculoskeletal problems. Many studies confirm the presence of these burdens and the necessity to undertake actions to minimize or eliminate them [25].

Our studies, burdens accompanying paramedics in their workplace were identified, resulting from assuming improper positions while performing procedures. The studies focused on analyzing the kinematics of the movement of individual body segments of the paramedic and muscle tension while performing standard medical tasks in the ambulance, both at a standstill and during driving. Medical procedures were performed by the paramedic on a medical simulator.

The study shows that typical medical procedures exceed normal movement ranges in the spine and hip joints (Table 7). Among the 31 measured angles, those concerning the spine and hips were selected. In the analyzed medical procedures, their normal movement range was exceeded due to the need for the paramedic to assume a forced position while performing medical activities. During the performance of medical activities, exceedances of normal ranges at the lumbar and thoracic levels occur, as confirmed by other studies [4,14,[26], [27], [28]]. The nature of the procedures often required, in addition to performing a given movement in each spine segment, also rotation in the hip joint, which is particularly visible in procedures P1, P3, and P4, i.e., lower limb immobilization, thermal isolation, and stopping bleeding from the lower limb. Essentially, performing each procedure required assuming a forced body position, and in each case, excessive rotational movements occurred. These results indicate the possibility of musculoskeletal system ailments among medical workers who perform such activities.

Therefore, it can be hypothesized that each of the procedures analyzed in this article was accompanied by discomfort related to exceeding several or most of the movement ranges in the examined spine segments and the hip joint. Excessive muscle loads in the lumbar spine were also demonstrated during each procedure, with the smallest exceedances occurring in procedure P2. The occurrence of both phenomena may result in musculoskeletal system ailments.

Both the own research and the review of publications confirmed the significant importance of ergonomic factors during the performance of medical procedures by paramedics, which may lead to an increased risk of injuries or accidents. The determination of the degree of fatigue resulting from excessive muscle tensions (Table 8) is influenced by the time of maintaining these positions, as well as the repetitiveness of the procedures during the shift. In 2022, EMS members in the eastern Mazovia region performed over 300,000 procedures, 20 % of which were carried out on trauma patients. Additionally, muscle tension may also result from the need to ensure body stabilization while performing medical activities on the scene, in the stationary ambulance, and during patient transport. The resulting musculoskeletal injuries increase the risk of long-term effects in the form of degenerative spine diseases, leading to prolonged absence from work [15,16]. The results of the own research indicate that factors analyzed in our study significantly contribute to the development of musculoskeletal disorders in paramedics. Similar problems also occur in EMS systems in other countries. Eiche et al. [5] conducted studies in Germany indicating that musculoskeletal system overload is common among paramedics, leading to increased employee turnover and job dissatisfaction. Similarly, studies Friedenberg et al. [29] confirm that these overloads result from the need to assume uncomfortable positions during resuscitation and patient transport. Paramedics often suffer from musculoskeletal injuries while providing medical assistance to trauma patients due to lifting and carrying patients or medical equipment [3,6,26,30]. The high rate of injuries among medical personnel providing assistance in Emergency Medical Teams is demonstrated in numerous studies both Polish and foreign [2,3,[31], [32], [33]]. Musculoskeletal disorders are one of the most common causes of work absence [6,34]. Pain. discomfort. and poor well-being caused by musculoskeletal system overload during work affect paramedics daily. The causes of such symptoms are manifold. Most often, musculoskeletal ailments result from prolonged maintenance of inappropriate, highly stressful, static body positions assumed during medical procedures, lifting, and carrying patients or medical equipment while providing assistance to trauma patients [4,24,[35], [36], [37], [38]]. Assuming uncomfortable body positions for extended periods, even many hours during work, can cause pain, mood deterioration, and increased susceptibility to injuries [39]. Performing medical procedures in forced, often uncomfortable body positions can lead to micro-injuries of the spine, resulting in complications in the form of chronic overloads and degenerative changes within this anatomical structure [28]. Based on a survey conducted among 120 paramedics working in emergency departments in the Lublin region (Poland), respondents indicated back pain (11.7 %) as the main reason for seeking medical advice [14]. In a study conducted in the Wielkopolska region (Poland). the occurrence of spine pain syndrome among all surveyed paramedics over the past 12 months was assessed. The study was conducted among 70 paramedics aged 24–56 years, working in a shift system. Most respondents (62 %) declared that pain occurs at most a few times a month. In 17 % of respondents, ailments appeared no more than once a week, while in 13 % of respondents, several times a week. Only 8 % suffered from back pain daily. In 64 % of paramedics, pain was mainly localized in the lumbosacral region, followed by the cervical (20 %) and thoracic (16 %) regions. It is worth noting that before taking up the profession of paramedic, most (94 %) did not experience back pain. Respondents identified lifting (31 %), maintaining a forced position (23 %), forward bending of the trunk (16 %), prolonged standing (9 %), and sitting (6 %), lifting weights (7 %), twisting the trunk (5 %), or excessive stretching (3 %) as the main causes of deterioration [28]. Moreover, due to the nature of their work, paramedics are exposed to a range of factors that can directly or indirectly affect the risk of workplace accidents [40]. Studies on paramedics indicate that this is a professional group exposed to a range of physical hazards that can lead to severe injuries or even death. In Australia, this risk was six times higher than in other occupational groups, and in the United States, mortality among paramedics during medical activities was over twice as high compared to other professions [41].

Alson et al. [19] showed that procedures such as limb immobilization and bleeding control are particularly physically demanding for paramedics. Our results confirm these observations, specifically showing increased muscle activity and joint overloads during these procedures, suggesting the need for further research on optimizing the ergonomics of paramedics’ work.

Thus, our observations, confirming the high burden on the musculoskeletal system during typical procedures in the ambulance, are consistent with the results of these studies. Moreover, research conducted by Friedenberg et al. [29] showed that 30 %–65 % of paramedics experienced back pain in the past year due to working in the confined spaces of ambulances. The results of our own research indicate similar proportions among the surveyed paramedics, indicating the global nature of this problem.

It should be noted that our study was conducted for 17 procedures, of which the analysis of five is presented in the article. The obtained results should serve as a starting point for a detailed analysis of the working positions assumed by paramedics during the activities performed in each procedure in terms of work organization, the arrangement of equipment and medical supplies, and the spatial structure of the ambulance. It is also worth examining the duration of recorded positions of paramedics during procedures and their repetitiveness. A comprehensive analysis of these factors will help identify the most burdensome positions, reduce their frequency and duration, or replace them with safer alternatives, thereby reducing the number of injuries and accidents among paramedics.

This pilot study identified forced positions during paramedic procedures. The research was conducted both at a standstill and during the movement of the ambulance for typical 17 procedures to identify the most strenuous ones in terms of work position.

The article presents the results obtained for 5 procedures that are most commonly performed when assisting trauma patients, both in terms of the range of motion in joints and the muscle tension involved. The study did not consider the anthropometric characteristics of the paramedic. However, the population of individuals performing this profession includes persons in the 5th to 95th percentile, of both sexes.

The anthropometric dimensions of the paramedic can influence the comfort of performing procedures, which use various equipment and medical materials located in specific places within the spatial structure of the ambulance cabin. The results of this study serve as a starting point for further actions in designing ergonomic interiors of ambulances and increasing the work comfort for paramedics, thereby reducing the risk of work-related injuries. The primary limitation of this study is the use of a single participant, which constrains the generalizability of the findings. While the paramedic included in the study was highly experienced and representative of typical ambulance working conditions, the lack of variability in participants means that the results may not account for differences in anthropometric characteristics, experience levels, or ergonomic challenges faced by paramedics of different body types.

To address this, future studies will involve a larger sample size, including paramedics of various ages, genders, and anthropometric profiles. This will enable more robust statistical analyses and provide greater insight into the variability of musculoskeletal loads during ambulance procedures.

The stretchers used in this study were not height-adjustable, which represents a limitation of the study. A height-adjustable stretcher would likely reduce the strain on paramedics by allowing for more ergonomic positioning during medical procedures. Future studies should consider incorporating height-adjustable stretchers to more accurately assess the impact of stretcher height on musculoskeletal load and ergonomic outcomes.

## 5 Conclusions

The study shows musculoskeletal overloads from forced positions during ambulance tasks. Therefore, it can be concluded that the risk of developing musculoskeletal disorders among paramedics is high, which is confirmed by available studies conducted in this professional group. This study provides preliminary findings to guide the development of methods for assessing ambulance ergonomics. The developed method will allow for the identification of sources of strain and the design of an ergonomic ambulance interior, ensuring comfort and reliability for emergency medical teams, and reducing the risk of injuries during medical procedures in the ambulance. This pilot study provides valuable preliminary insights into the ergonomic challenges faced by paramedics. However, to fully validate these findings, larger-scale studies are planned with diverse participant groups. These will allow for statistical analyses of differences in musculoskeletal strain across procedures and participants, ultimately guiding evidence-based recommendations for ambulance design and operational practices. As a result of the conducted research, several sources of discomfort were identified that force paramedics to assume constrained postures while performing medical procedures. These include.

• External load caused by the weight of the patient's limb.
• Improper arrangement of materials necessary for the procedure in the ambulance cabin compartments.
• Lack of a table to hold materials needed during the procedure.
• Stretchers that are too low (lack of height adjustment), requiring paramedics to bend over the patient.
• Access to the patient limited to one side and the head area.
• Inability to perform the procedure while seated.

Minor modifications to the ambulance interior can be introduced during its operational use. For example, in the Meditrans Emergency Medical Services ambulances in Siedlce, safety nets were installed to secure paramedics performing procedures while standing during ambulance travel. However, the optimal stage for implementing such changes is during the prototype phase, which is the focus of our ongoing research.

Future studies on a larger group of paramedics, accounting for anthropometric variability and gender differences, and including additional procedures, will allow for the development of recommendations for modifying the spatial layout of ambulance cabins. The primary objective of this comprehensive research is to achieve standardization across ambulances of the same type, which will significantly enhance the efficiency and comfort of paramedic work.

Future studies should not only assess the physical and physiological aspects of paramedic work but also include an evaluation of workplace stress. Understanding the impact of stress on paramedic performance and its interaction with musculoskeletal load could lead to more comprehensive recommendations for improving both the physical and mental well-being of emergency medical workers.

## CRediT authorship contribution statement

**Daniel Celiński:** Writing – review & editing, Writing – original draft, Data curation. **Sylwia Bęczkowska:** Writing – review & editing, Writing – original draft, Validation, Supervision, Methodology, Formal analysis, Data curation, Conceptualization. **Iwona Grabarek:** Writing – review & editing, Writing – original draft, Validation, Supervision, Methodology, Formal analysis, Data curation, Conceptualization. **Katarzyna Grzybowska:** Writing – review & editing, Writing – original draft, Validation, Supervision, Methodology, Formal analysis, Data curation, Conceptualization. **Zuzanna Zysk:** Writing – review & editing, Writing – original draft, Validation, Supervision, Methodology, Formal analysis, Data curation, Conceptualization. **Tadeusz Miłowski:** Writing – review & editing, Conceptualization. **Krzysztof Marek Mitura:** Writing – review & editing, Writing – original draft, Data curation, Conceptualization. **Sławomir Dariusz Szajda:** Writing – review & editing, Writing – original draft, Conceptualization.

## Ethics declaration

The study (9/2022) was conducted with the approval of the Research Ethics Committee of the Warsaw University of Technology on November 23, 2022.

## Financial disclosure

This research did not receive any specific grant from funding agencies in the public, commercial, or not-for-profit sectors.

## Declaration of competing interest

The authors declare that they have no known competing financial interests or personal relationships that could have appeared to influence the work reported in this paper.
